# Environmental influences on the skin microbiome of humans and cattle in rural Madagascar

**DOI:** 10.1093/emph/eox013

**Published:** 2017-08-26

**Authors:** Melissa B. Manus, James J. Yu, Lawrence P. Park, Olaf Mueller, Sarah C. Windsor, Julie E. Horvath, Charles L. Nunn

**Affiliations:** 1 Duke Global Health Institute, Duke University, Durham, NC, USA; 2 Department of Evolutionary Anthropology, Duke University, Durham, NC, USA; 3 Department of Medicine, Duke University School of Medicine, Durham, NC, USA; 4 Department of Molecular Genetics and Microbiology, Duke University, Durham, NC, USA; 5 Center for the Genomics of Microbial Systems, Duke University School of Medicine, Durham, NC, USA; 6 North Carolina Museum of Natural Sciences, Raleigh, NC, USA; 7 Department of Biological and Biomedical Sciences, North Carolina Central University, Durham, NC, USA

**Keywords:** microbiome, skin, cattle, environment, mismatch, Madagascar

## Abstract

**Background and objectives:**

The skin harbors a dynamic community of microorganisms, where contact with humans, other animals and the environment can alter microbial communities. Most research on the human skin microbiome features Western populations living in hygienic conditions, yet these populations have vastly different patterns of environmental contact than the majority of people on Earth, including those living in developing countries.

**Methodology:**

We studied skin microbial communities of humans and cattle (zebu) in rural Madagascar to investigate how zebu ownership affects microbial composition of the human skin, and to characterize non-Western human and zebu skin communities more generally. A portion of the 16S rRNA gene was sequenced from samples of zebu backs and human ankles, forearms, hands and armpits. Analyses were conducted in QIIME, R and LEfSe.

**Results:**

Human and zebu samples varied in microbial community composition, yet we did not find evidence for a shared microbial signature between an individual and his zebu. Microbial communities differed across human body sites, with ankles reflecting increased diversity and greater similarity to samples from zebu, likely due to extensive shared contact with soil by humans and zebu.

**Conclusions and implications:**

Cattle ownership had, at best, weak effects on the human skin microbiome. We suggest that components of human biology and lifestyles override the microbial signature of close contact with zebu, including genetic factors and human–human interaction, irrespective of zebu ownership. Understanding ecological drivers of microbial communities will help determine ways that microbial transfer and community composition change as populations adopt Western lifestyles, and could provide insights into zoonotic disease transmission.

## INTRODUCTION

Ecological concepts such as dispersal, species diversity and community assembly help to describe the human body as an interactive ecosystem in which health outcomes are a type of ecosystem service to the host that is influenced by microbes [1]. Different body sites harbor distinctive communities of microbes based on the properties of that particular skin ecosystem [2, 3], including the local temperature, moisture level and pH [4]. Because the skin is constantly exposed to the outside world, contact with the environment can alter the composition of the microorganisms that live on it [5]. For example, skin microbes are deposited directly on contacted surfaces [6] and disperse to new individuals via these surfaces and by human-to-human skin interaction [7]. These community-level changes can perturb the overall ecosystem to induce disease states, even in the absence of new pathogen invasion [4, 8].

Because contact with shared surfaces is known to homogenize skin communities, it is likely that human skin is also affected by the microbes of animals with which they come into contact [9]. Domesticated animals are of particular interest, as they come into regular contact with and often live in close proximity to owners. Previous studies on Western populations have shown that the skin microbial communities of dog owners are more similar to the communities of their dogs than to those of other dogs, and that close contact with dogs significantly influences the microbial communities on the human hand [9, 10].

Despite the recent increase in microbiome research, much of this effort has focused on Western populations and involved laboratory cultures for detecting pathogen transmission by particular microbial species [2, 7, 11]. Expanding the breadth of microbiome research to include non-Western populations and domesticated animals can inform understanding of microbe-related health outcomes on a global scale [2, 12]. Because rural, non-Western populations are more closely connected to the ancestral human environment, data from these populations can provide insights into how modern environments are linked to health conditions. These data are also relevant to the field of evolutionary medicine, and specifically to *evolutionary mismatch*, the recognition that differences between the current environment and the one in which humans evolved have direct consequences for our health [13]. Characterization of the skin microbiome in non-Western populations with more traditional lifestyles is an important step in determining whether changes in environmental contact are generating conditions for evolutionary mismatches that are relevant to disease [12].

We aimed to assess the influence of environmental contact on the composition of skin microbial communities among individuals living in rural Madagascar, where people interact closely with an environment shared by cattle, chickens, pigs and other domesticated animals. We focused especially on contact with zebu (*Bos taurus indicus*)—the domesticated cattle of Madagascar—as these animals are commonly used for heavy work (e.g. plowing fields). This results in contact between zebu and their human owners, especially as humans physically manipulate the animals while working in the fields. In this setting, zebu owners spend a significant portion of the day with their zebu, providing ample opportunity for physical contact in a shared environment.

We hypothesized that contact with zebu influences the skin microbial community of people who regularly interact with zebu (hereafter, referred to as zebu owners). We predicted that there would be differences between the skin microbial communities of zebu owners and those who do not own zebu (i.e. non-owners). Because of increased contact between zebu owners and zebu, we predicted that a given zebu’s skin microbiota would be more similar to its owner than to other owners who also own zebu. Microbial dispersal from the shared environment of zebu owners and zebu may also lead to community differences among body sites. Thus, we sampled four human body sites and compared them to zebu, as previously conducted in studies of Western populations and other domesticated animals [14, 15]. Because zebu owners are often barefoot while working in the fields, providing a point of regular connection between human and zebu via the ground, we expected a zebu’s skin microbiota would be more similar to its owner’s ankle than to other body parts.

A lack of support for these predictions could be consistent with at least two alternative hypotheses. First, factors intrinsic to humans and zebu, including genetics, could create ecological conditions that favor the colonization of particular communities of microorganisms, with little or no influence of the lifestyle factors associated with zebu ownership. Second, the scale of human contact with zebu may be dwarfed by substantially greater contact with other humans in settings that exclude zebu (i.e. in the home, with neighbors), resulting in a weaker signal of zebu ownership. Both alternatives would result in fewer differences between the microbial communities of the human groups (zebu owners and non-owners) than between communities of zebu owners and zebu.

## METHODS

### Setting

Data collection took place in Mandena (∼-18°42′00″ S 47°50′00″ E), a village of ∼3000 people in the SAVA region of Madagascar (an acronym capturing the names of its four major cities, Sambava, Antalaha, Vohémar and Andapa). Agricultural practices and adjacency to Marojejy National Park drive interactions among humans, wildlife, domesticated animals and the environment. With temperate conditions and ample rainfall, Mandena relies on the production of rice and vanilla, and many individuals use zebu to work in the rice fields.

All study procedures were approved by Duke University’s Institutional Review Board (Protocol C0848) and Institutional Animal Care & Use Committee (Protocol A097-15-03), and by Malagasy health authorities. In-country permits were obtained with the help of the Madagascar Institute for the Conservation of 

Tropical Environments (MICET), a non-governmental organization that acts as a liaison between the Malagasy government and foreign researchers. All sample transportation was approved by the United States Department of Agriculture, Animal and Plant Health Inspection Service (permit number 127958).

### Participants

The study included 20 adult males (defined as eighteen years of age or older) living in Mandena. Participants ranged from 18 years to 75 years of age and were initially recruited through a meeting officiated by the village president at a central building in Mandena. Interested individuals were asked to return to the building on specific dates. After obtaining informed consent with the help of a Malagasy translator, individuals completed a general health survey to screen for eligibility in the skin microbiome study. Each participant was assigned an anonymous identification number, which was kept throughout the study. Basic health measurements were taken, including temperature, blood pressure, heart rate, height and weight. Individuals with abnormal clinical measurements or open wounds were ineligible for the study and were directed to speak with the Malagasy nurse working with the research team about healthcare options. All participants were compensated with a fresh coconut for completing the general health survey.

We recruited ten adult males who owned and regularly worked with zebu and ten adult males who do not come in regular contact with zebu. ‘Regular contact’ was assessed using questions on the general health survey, and in the context of this village, was synonymous with an individual reporting that his occupation is ‘farmer.’ If a zebu owner was eligible for the study, his animal was automatically enrolled; if an individual had more than one animal, we selected the adult animal that was reported to be used most consistently in the fields. During this initial recruitment period, >20 adult males expressed interest and took the general health survey. We continued to administer surveys, but no longer recruited participants after the first 20 eligible individuals were identified. Participants were given a piece of paper with the date to return for their sample collection. Individuals were encouraged to return to the central building for sample collection, although logistical constraints of the long workdays in the field often required sample collection to take place at the individual’s home or in the agricultural fields, especially for participants who owned zebu.

### Sample collection

When a participant returned for sample collection, he was guided through a second informed consent process and short survey with the help of a Malagasy translator. Zebu owners were informed of how samples would be collected from their animals. Skin swab samples were obtained using a sterile, dual-tipped rayon swab (Fisher BD BBL CultureSwab, Media-free, manufacturer number 220135). The swab was rubbed vigorously over the dry sample site for 30 s. Authors MBM and JJY collected all samples. To establish trust and make the participant comfortable, samples were taken in a specific order, from least to most invasive: (i) back of right hand, (ii) inside of right forearm, (iii) right ankle and top of the foot and (iv) right armpit. These body sites were chosen to represent ways in which a person likely contacts his zebu and the environment. In particular, hands and arms are used to handle the zebu in the fields, and people are often barefoot while working alongside their animals, resulting in frequent environmental contact for these body sites. In contrast, the armpit comes into less contact with the outside environment than do the other sites.

To control for variables that might influence the skin microbiome, such as temperature and rainfall, zebu samples were collected as quickly as possible after collecting its owner’s sample. Zebu samples were collected from behind the dorsal hump, an area that was frequently touched by human arms and hands. Identical swabbing techniques were used on both humans and zebu. All human participants were compensated with two bars of Santex soap, a highly valued yet not readily accessible product, at the end of the study.

Conditions in the field site and during transport made it impossible to maintain the samples in sub-freezing conditions. A previous study found that differences in storage conditions near room temperature do not significantly affect microbial community composition [16], while the impacts of freeze–thaw cycles are unstudied. Thus, it was determined that keeping the samples uniformly cool was preferable to subjecting the samples to repeated (and unpredictable) freeze–thaw cycles. Mandena is not supported by electricity, but a small refrigerator powered by a generator was used to store the samples in the village when it was available. Otherwise, samples were kept at room temperature, at a daytime mean of 20°C (David Samson, personal communication, 2017), inside a plastic cooler that was stored inside a dark room. Samples were collected over two sampling periods (the first from July 12 to 14, 2015 and the second from July 20 to 21, 2015) and returned to the United States within two weeks of collection, on two separate flights (in mid and late July 2015). Refrigeration was used in hotels during transit, and samples were stored in coolers, typically with freezer packs or ice, during ground transportation. On airplanes, the samples were placed in insulated envelopes that included freezer packs.

### Laboratory procedures

Upon arrival in the US, samples were stored long-term at -80°C at the North Carolina Museum of Natural Sciences in Raleigh, North Carolina. DNA was extracted from each swab using the MOBIO PowerSoil DNA Isolation kit (MO BIO Laboratories, Inc., Carlsbad, CA, USA). The Electronic Supplementary Materials detail modifications to the DNA extraction protocol.

Following DNA isolation, the samples were transferred to the sequencing facility at Duke University’s Center for Genomic and Computational Biology, where forward and reverse primers (F-5′TCGTCGGCAGCGTCAGATGTGTATAAGAGACAGCCTACGGGNGGCWGCAG and R-5′ GTCTCGTGGGCTCGGAGATGTGTATAAGAGACAGGACTACHVGGGTATCTAATCC were used to amplify the V3-V4 regions of the 16S rRNA gene, and amplicons were sequenced using the Illumina MiSeq platform [17, 18]. Complete library preparation can be found in the Electronic Supplementary Materials. There were 44 308 999 MiSeq read pairs joined in QIIME (Quantitative Insights into Microbial Ecology, 1.9.1) [19], with join_paired_ends.py, using the fastq-join method with a maximum percentage difference (-*P*) of 25%. Assembled amplicons were further quality filtered with split_libraries_fastq.py (phred quality Q20), resulting in 20 583 303 sequences used for downstream analysis. 16S rRNA Operational Taxonomic Units (OTUs) were picked using the closed reference method with SortMeRNA and the SILVA database (version 1.23) at 97% similarity [20, 21]. The majority of our analyses excluded bacterial species, as many taxa are difficult to distinguish at this level using a small portion of the 16S rRNA gene.

A minimal sampling depth (-*e*) of 8000, with default parameters, was used for subsequent diversity analyses with core_diversity_analyses.py of the QIIME package. This sampling depth was chosen in order to include all zebu samples in further analyzes. Rarefaction resulted in a total of 4 363 250 reads from 85 samples (2 621 460 reads from 75 human samples and 1 741 790 from 10 zebu samples).

Alpha diversity was measured for the four human sample sites and the single zebu sample site using Faith's phylogenetic diversity (PD), a quantitative measure of the sum of the branch lengths in a phylogenetic tree for a given set of taxa [22]. Beta diversity was measured using unweighted UniFrac community distance to measure the similarity of community structure of microbes within and between groups and sample sites. This method assesses similarities (presence/absence of taxa) across communities based on the proportion of branch length in a phylogenetic tree that is shared between two samples [23]. Unweighted UniFrac distances, which ignore relative abundance of taxa, were used instead of weighted UniFrac distances to avoid any bias introduced by storage method (i.e. the relative abundance of various taxa may change in different ways in cool, rather than freezing, conditions).

### Statistics

All statistical analyses were conducted in R version 3.2.3 [24]. Kruskal–Wallis tests were conducted for Faith’s PD and the number of OTUs on zebu, zebu owner and non-owner samples, using a critical alpha level of 0.05. Linear regression and linear mixed effect models were constructed using the stats and MuMIn packages, respectively [24, 25]. Unweighted UniFrac distances were used to generate Principal Coordinate Analysis (PCoA) plots and were utilized in the mixed effect models.

For many of our analyses, statistical methods were chosen to maximize predictions, avoid significance levels in the form of *P*-values, and ensure the use of all samples rather than limiting the analyses to subsets of data. Thus, using linear mixed effect models, we performed model selection [26] to: (i) assess the difference in microbial community composition (i.e. UniFrac distance) between zebu owners and zebu, compared with zebu owners and non-owners; (ii) test whether a zebu is more similar to its owner than to other zebu owners and (iii) test whether a zebu is more similar to its owner’s ankle than to its owner’s armpit. For these models, smaller UniFrac distances indicate shorter distances between *x* and *y* when plotted on a PCoA, and thus, fewer community differences. We selected the model with the lowest Akaike Information Criteria (AIC) score, correcting for small sample size (AIC_c_), and included individual ID as a random effect. We also used linear regression models to predict the number of OTUs on different body sites, and we used linear discriminant analysis effect size (LEfSE) analysis between zebu and humans to investigate differential abundance of particular microbial taxa in these groups [27].

## RESULTS

### Host differences

**Figure 1.**
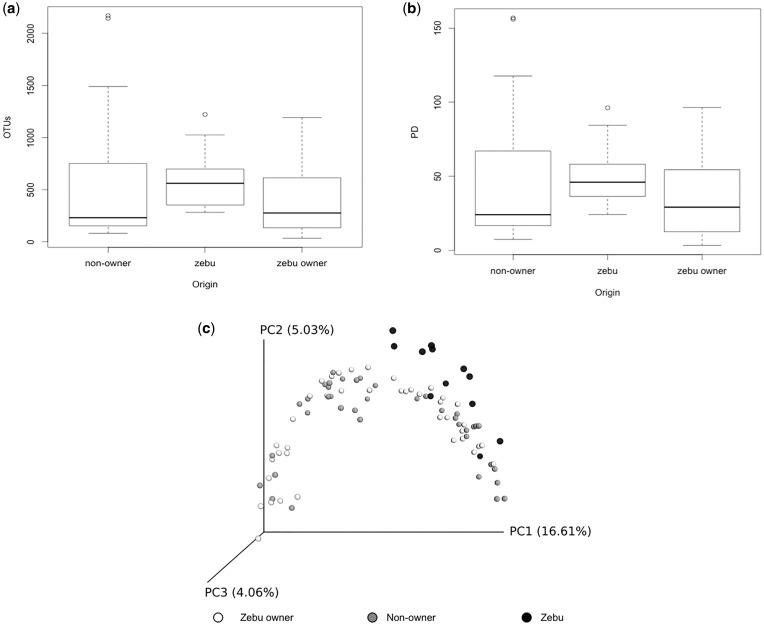
Comparison of alpha and beta diversity at the host level: (**a**) Boxplots of number of OTUs on zebu, zebu owners and non-owners (all body sites combined); (**b**) Boxplots of PD on zebu, zebu owners and non-owners (all body sites combined); (**c**) PCoA of unweighted UniFrac community distances of microbes between zebu (black), zebu owners (white) and non-owners (gray)

When comparing zebu owners, non-owners and zebu, we found no significant difference in number of OTUs (Kruskal–Wallis chi-squared = 4.6, d.f. = 2, *P *= 0.1, Fig. 1a) or PD (Kruskal–Wallis chi-squared = 3.9, d.f. = 2, *P* = 0.1, Fig. 1b). In terms of beta diversity, the zebu samples clustered apart from both human groups on a PCoA of unweighted UniFrac distances (Fig. 1c). However, the two human groups did not segregate from each other.

**Figure 2.**
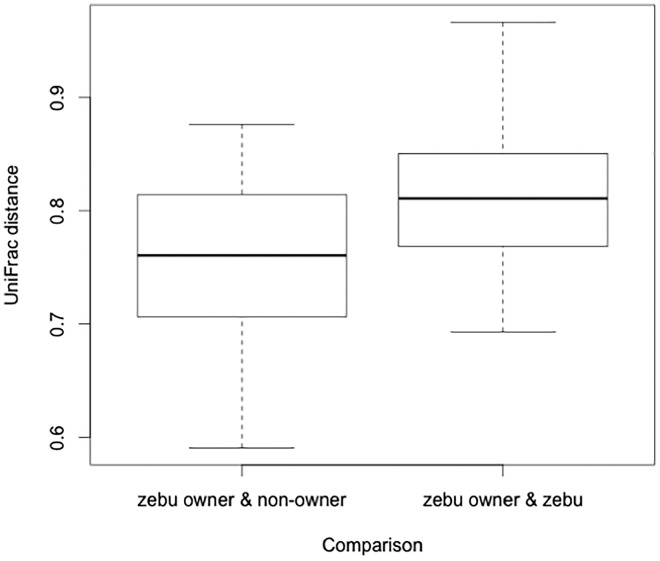
The unweighted UniFrac distance between zebu owners and non-owners compared with zebu owners and zebu; zebu owners are more similar to non-owners than to zebu

Linear mixed effect models yielded similar results. Host comparison (zebu owner/zebu vs zebu owner/non-owner) had an effect on UniFrac distance (AIC_c_ = -162.4, d.f. = 4, weight = 0.920), though human body site did not (AIC_c_ delta = 5.02). The UniFrac distance was significantly larger between zebu owners and zebu than between zebu owners and non-owners (Fig. 2). Thus, zebu owners are more similar, in terms of their skin microbial communities, to non-owners than to zebu.

**Figure 3.**
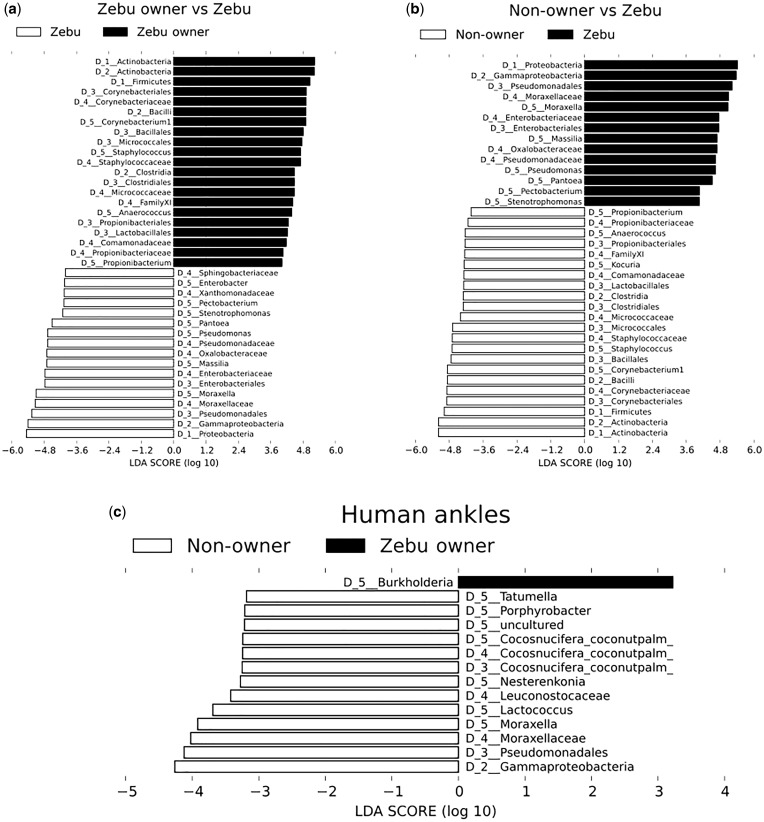
Top discriminative taxa as determined by LEfSe analysis: (**a**) zebu owners (all body sites combined) and zebu; (**b**) non-owners (all body sites combined) and zebu; (**c**) zebu owner ankles and non-owner ankles

Ankle samples of zebu owners and non-owners were composed of similar proportions of Proteobacteria, Actinobacteria, Bacteroidetes, Firmicutes (the four most common human skin bacterial phyla) [4] and Cyanobacteria, a diverse phylum of aquatic bacterium that include species capable of producing toxins that have been shown to affect human and non-human animal health [28, 29] (Supplementary Fig. S1b and d). Proportions of these taxa in armpit samples were also similar between the two groups (Supplementary Fig. S1a and c). In contrast, zebu samples were dominated by Proteobacteria (88.5%) (Supplementary Fig. S1e). Zebu owner samples had higher proportions of Actinobacteria and Firmicutes than did zebu samples (Fig. 3a;Supplementary Fig. S1c–e). Complete LEfSe analysis showing microbial composition differences between zebu, zebu owners and non-owners can be seen in Fig. 3a–c.

### Sample site differences

**Figure 4.**
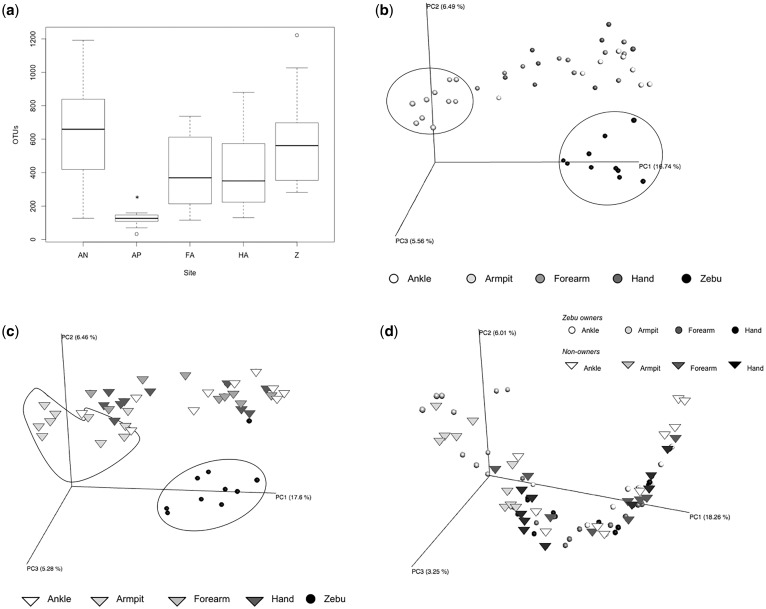
Comparison of alpha and beta diversity at the level of body site: (**a**) Number of OTUs on zebu owner body sites compared to zebu (*armpits are significantly different from all other body sites; AN = ankle, AP = armpit, FA = forearm, HA = hand, Z = zebu); (**b**) PCoA of unweighted UniFrac distance of microbes in zebu owners and zebu (groups of armpit and zebu samples are outlined); (**c**) PCoA of unweighted UniFrac distance of microbes in non-owners (triangles) and zebu (circles) (groups of armpit and zebu samples are outlined); (**d**) PCoA of unweighted UniFrac distance of microbes in zebu owners (circles) and non-owners (triangles)

The armpit was the least diverse human body site (Fig. 4a;Table 1). In a linear model that used zebu samples as the reference, zebu owner armpits contained significantly fewer OTUs, while non-owner ankles contained significantly more OTUs (Table 1). PCoAs of unweighted UniFrac distances revealed that armpit samples cluster distinctly from zebu samples and samples from other human body sites (Fig. 4b–d).

**Table 1.** Linear model predicting number of OTUs on zebu owners and non-owners, using zebu as reference

	Body site	Estimate	Lower CI	Upper CI	*t*-Value
Zebu owners	Armpit	-420.34	-721.16	-119.51	-3.35
	Forearm	-26.03	-259.44	207.38	-0.24
	Hand	-125.22	-454.61	204.18	-0.63
	Ankle	105.95	-223.45	435.34	0.89
Non-owners	Armpit	-176.35	-467.41	114.71	-1.21
	Forearm	125.94	-178.11	429.99	0.82
	Hand	35.81	-255.25	326.87	0.25
	Ankle	440.77	149.71	731.83	3.01

Zebu owner armpit samples contain significantly fewer OTUs than do zebu samples, and non-owner ankle samples harbor significantly more OTUs than do zebu samples.

While Proteobacteria dominated the zebu samples, human ankle samples contained only roughly one-third of this taxon, while human armpit samples harbored <10% (Supplementary Fig. S1b, d, e). The frequency of Actinobacteria was almost equal in the armpit and ankle samples, while the frequency of Firmicutes was more than doubled on armpits compared with ankles (in both zebu owners and non-owners) (Supplementary Fig. S1a–d). Bacteroidetes was found in similar proportions across ankles and armpits, while Cyanobacteria was found on ankles, yet was virtually absent from armpit samples.

LEfSe analysis revealed differences between ankle samples of zebu owners and non-owners to be driven by a number of taxa (Fig. 3b). Notably, zebu owner ankles contained more *Burkholderia*, while non-owner ankles contained more Gammaproteobacteria. Both of these taxa include potentially pathogenic organisms [30, 31].

### Similarity of zebu and zebu owners

We investigated the similarity between a zebu and its owner using linear mixed effect models to predict UniFrac distances, where smaller distances indicate more community similarity between groups of samples. Including host comparison (zebu/its owner vs zebu/other owners) did not improve prediction of UniFrac distance in a model using owners’ ankles (AIC_c_ delta = 2.27) or in a model using owners’ forearms AIC_c_ delta = 2.15). Contrary to our prediction, this indicates that microbial communities of zebu were no more similar to those of their owners than to those of other zebu owners.

**Figure 5.**
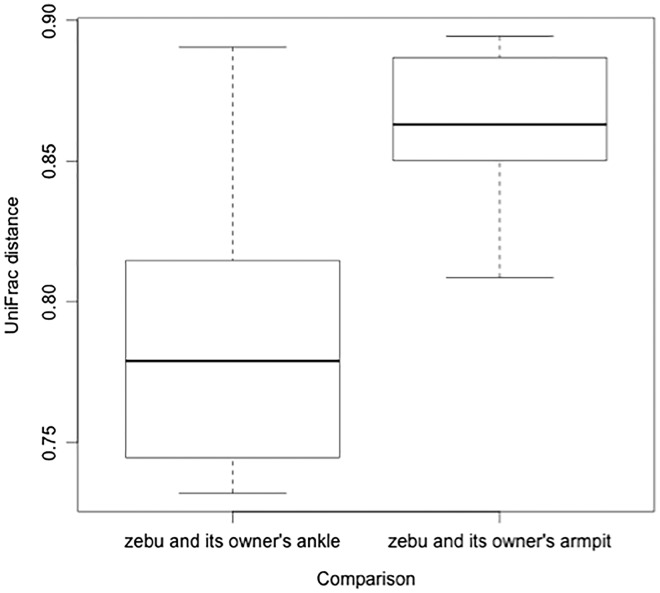
The unweighted UniFrac distance between a zebu and its owner’s ankle compared to the same zebu and its owner’s armpit; zebu are more similar to owner ankles than to owner armpits

In contrast, body site was predictive of UniFrac distance in a model that compared a zebu and its owner’s armpit to a zebu and its owner’s ankle (AIC_c_ = -46.2, d.f. = 4, weight = 0.965). The distance between a zebu and its owner’s ankle was smaller than the distance between a zebu and its owner’s armpit, indicating that zebu skin microbial communities are more similar to those of their owner’s ankle than to those of their owner’s armpit (Fig. 5).

## DISCUSSION

We found no significant differences in microbial diversity between zebu owners and non-owners; instead, there were clearer distinctions in microbial communities across different human body sites. Our findings suggest that contact with zebu is not a major driver of skin microbial communities on zebu owners. Two possible alternative explanations are consistent with our findings, and are not exclusive of one another. First, there may be intrinsic host factors that drive differences in the microbial communities on humans compared with those on zebu. For example, certain bacterial taxa may be better suited to colonizing human skin than zebu skin, perhaps based on differences in hair, sweat glands, pH or host genetics [3]. Second, humans may have greater overlap in shared environments with other humans than they do with zebu. Our findings suggest that interactions within the shared environment of all humans, regardless of zebu ownership, can homogenize the skin microbiome, but that different body sites may harbor distinct microbial communities due to dispersal from environmental microbes, two observations previously reported [3, 9].

These two alternative scenarios are consistent with the hypothesis that humans carry a distinct microbial fingerprint [7], which was supported by the finding that inter-group (zebu to human) UniFrac distances were greater than intra-group (human to human) [7, 12]. Similar OTUs drive differences between zebu/zebu owners and zebu/non-owners, including those of Actinobacteria and Firmicutes (Fig. 3a and c), possibly indicating more successful colonization of human skin by these taxa. In contrast, Proteobacteria dominated zebu samples (89%), but made up only roughly one-third of human ankle communities (Supplementary Fig. S1b, d, e).

It is also important to consider potential behavioral drivers of differences across body sites. Armpit samples (regardless of zebu ownership) clustered apart from the other body sites on a PCoA, a trend observed previously [14]. Similarly, the microbial communities of zebu were less similar to those of zebu owner armpits than to those of zebu owner ankles. The microorganisms living in the armpit are more sheltered than are those in other body parts that we sampled, and would thus be less likely to have regular contact with environments shared between zebu and zebu owner ankles. Additionally, human armpits are unique in that they contain both eccrine and apocrine sweat glands; this likely contributes to the differential success of certain microbes that feed on the compounds produced in the armpit [32, 33].

Zebu owner ankles contained more *Burkholderia* than did non-owner ankles (Fig. 3b). This genus consists of human, animal and plant pathogens, as well as beneficial soil-derived species [30]. It would be interesting to determine if these taxa are transient members of the human skin community as a result of contact between humans and zebu, or if long-term contact with zebu (and the shared environment) results in fundamental shifts in human skin communities that allow taxa that are typically considered animal and plant microbes to become residents [3, 34].

We found that zebu samples contained more Gammaproteobacteria than did samples from humans, yet despite the connection to zebu, zebu owners harbored less of this taxon on their ankles than did non-owners (Fig. 3a–c). These findings support the idea that certain microbes originate from sources that are central to daily life in Mandena, independent of zebu ownership. This could include soil, river water, firewood, plants and other domesticated animals. Given the breadth of human and cattle pathogens in class Gammaproteobacteria, including *Helicobacter pylori*, *Rickettsia*, *Escherichia coli* and *Coxiella* (the bacterium responsible for Q-fever), understanding the biological and behavioral drivers of site-specific differences in harboring these taxa could have direct veterinary medicine, zoonotic disease and public health implications [31, 35].

Our results are similar to those found in other non-Western populations. A microbial signature of regular environmental contact with human skin was observed in both Amerindian and Tanzanian populations and may explain why the microbiome of a zebu is more similar to his owner’s ankle microbiome than to his owner’s armpit microbiome (Fig. 5) [11, 34]. Because neither the armpit nor the ankle is likely to come into direct contact with zebu, it is plausible that similarities that we documented between ankles and zebu are driven by elements of the shared environment. In the future, it would be interesting to compare lower legs of humans and zebu. The shared terrestrial environment of these sites could potentially result in stronger similarity than we observed with our data, and may include zoonotic transfer of pathogenic microbes, specifically through water, soil and zebu feces.

However, despite previous studies reporting similarities between the microbiomes of dogs and their owners in Western populations, a zebu was no more similar to its owner than to other zebu owners [9, 10]. This is likely due to the overwhelming effect of the shared environment, as well as homogeneity of Mandena households in relation to environmental exposure. Previous findings of pet–owner similarity in Western populations may reflect additional factors that drive differences in the built environments of each pet–owner pair. Not only do many domesticated animals in Western populations live inside the home with the owner, but also household-specific factors such as cleaning products, air conditioning units, open windows and clothing act to increase heterogeneity across each pet–owner pair’s environment. Variation in the pet–owner pair’s interaction with their individual home environments would further accentuate similarities between the pair. In contrast, we expect less variability among households in Mandena, as all homes are constructed from wood and plant materials, and lack of effective insulation or air conditioning may result in less distinction between individual homes and the broader, shared environment. The increased variability between pet–owner pairs may thus be another distinctive feature of Western lifestyles, as compared with people living in more rural settings.

A limitation of this study is its relatively small sample size. To deal with this weakness, we focused the majority of analyses on linear mixed models and avoided methods that rely on null hypothesis testing. For some models, we were able to increase the sample size to closer to 90, by including samples from all four human body sites in addition to the samples from zebu, using random effects to control for individual. While future studies would benefit from larger sample sizes, we would expect the direction of these patterns to remain unchanged (for example, human armpits contain fewer OTUs than do zebu; the UniFrac distance between a zebu and its owner’s ankle is smaller than the distance between a zebu and its owner’s armpit).

Future research should aim to strengthen our understanding of how deviations from ancestral lifestyles impact human, wildlife and environmental health, as a central part of the emerging field of evolutionary medicine. Aspects of Western lifestyle and the built environment may alter the way in which humans and domesticated animals interact with microbes [11]. Understanding these mismatches with regard to humans and cattle is of particular interest, as this relationship has existed in Madagascar since the twelfth century, and continues to be critically important for agriculture, wealth and status in human populations across the world [36]. Continued work with this Malagasy population would benefit from experimentally testing items such as socks and shoes for their inhibition of microbial sharing. This would help to quantify the effects of a Western lifestyle on the skin microbiome.

To the best of our knowledge, this is the first study to analyze the skin microbiome of the Malagasy people and their zebu, and one of only a handful that has investigated the skin microbiome in a non-Western, rural population [11, 12, 34]. This work contributes to the limited number of studies that have focused on non-Western populations, despite the pre-dominance of these populations in other global public health endeavors. Moving forward, clear connections between microbe communities and disease states will help inform medical and public health perspectives within the context of the human microbiome.

## SUPPLEMENTARY DATA

Supplementary data are available at *EMPH* online.

## FUNDING

This research was supported by the Duke Global Health Institute and the Bass Connections Program at Duke University.

**Conflict of interest**: None declared.

## Supplementary Material

Supplementary Data 1

Supplementary Figure_Legends

eox013_Supp_Figure1a

eox013_Supp_Figure1b

eox013_Supp_Figure1c

eox013_Supp_Figure1d

eox013_Supp_Figure1e

Supplementary Data 2
