# Supplementary material for: Environmental influences on the skin microbiome of humans and cattle in rural Madagascar
Source: Evol Med Public Health. 2017 Aug 26;2017(1):144–53. doi: 10.1093/emph/eox013 (PMC5631097; doi:10.1093/emph/eox013)
Supplement: Supplementary Data 1 [file eox013_Supp.docx]

S2 – DNA extraction protocol

*Standard laboratory procedure for DNA extraction using MoBio PowerSoil DNA Isolation kit. The following procedure was taken directly from MoBio, with modifications noted.*

1. To the PowerBead Tubes provided, add 0.25 grams of soil sample.

***Modification: the entire swab was submerged and mixed into the PowerBead Tube for 1 minute.***

2. Gently vortex to mix.

3. Check Solution C1. If Solution C1 is precipitated, heat solution to 60°C until dissolved before use.

***Modification: Solution C1 was heated at the start of Step 1.***

4. Add 60 μl of Solution C1 and invert several times or vortex briefly.

5. Secure PowerBead Tubes horizontally using the MO BIO Vortex Adapter tube holder for the vortex (MO BIO Catalog# 13000-V1-24) or secure tubes horizontally on a flat-bed vortex pad with tape. Vortex at maximum speed for 10 minutes. Note: If you are using the 24 place Vortex Adapter for more than 12 preps, increase the vortex time by 5-10 minutes.

6. Make sure the PowerBead Tubes rotate freely in your centrifuge without rubbing. Centrifuge tubes at 10,000 x g for 30 seconds at room temperature. CAUTION: Be sure not to exceed 10,000 x g or tubes may break.

7. Transfer the supernatant to a clean 2 ml Collection Tube (provided). Note: Expect between 400 to 500 μl of supernatant. Supernatant may still contain some soil particles.

8. Add 250 μl of Solution C2 and vortex for 5 seconds. Incubate at 4°C for 5 minutes.

9. Centrifuge the tubes at room temperature for 1 minute at 10,000 x g.

10. Avoiding the pellet, transfer up to, but no more than, 600 μl of supernatant to a clean 2 ml Collection Tube (provided).

11. Add 200 μl of Solution C3 and vortex briefly. Incubate at 4°C for 5 minutes.

12. Centrifuge the tubes at room temperature for 1 minute at 10,000 x g.

13. Avoiding the pellet, transfer up to, but no more than, 750 μl of supernatant into a clean 2 ml Collection Tube (provided).

14. Shake to mix Solution C4 before use. Add 1200 μl of Solution C4 to the supernatant and vortex for 5 seconds.

15. Load approximately 675 μl onto a Spin Filter and centrifuge at 10,000 x g for 1 minute at room temperature. Discard the flow through and add an additional 675 μl of supernatant to the Spin Filter and centrifuge at 10,000 x g for 1 minute at room temperature. Load the remaining supernatant onto the Spin Filter and centrifuge at 10,000 x g for 1 minute at room temperature. Note: A total of three loads for each sample processed are required.

16. Add 500 μl of Solution C5 and centrifuge at room temperature for 30 seconds at 10,000 x g.

***Modification: Solution C6 was heated at the start of this step.***

17. Discard the flow through.

18. Centrifuge again at room temperature for 1 minute at 10,000 x g.

19. Carefully place spin filter in a clean 2 ml Collection Tube (provided). Avoid splashing any Solution C5 onto the Spin Filter.

20. Add 100 μl of Solution C6 to the center of the white filter membrane. Alternatively, sterile DNA-Free PCR Grade Water may be used for elution from the silica Spin Filter membrane at this step (MO BIO Catalog# 17000-10).

***Modification: add 25 μl of Solution C6 and centrifuge for 30 seconds at 10,000 x g. Repeat with an additional 25 μl.***

21. Centrifuge at room temperature for 30 seconds at 10,000 x g.

22. Discard the Spin Filter. The DNA in the tube is now ready for any downstream application.
