# Supplementary figures and images for: Environmental influences on the skin microbiome of humans and cattle in rural Madagascar
Source: Evol Med Public Health. 2017 Aug 26;2017(1):144–53. doi: 10.1093/emph/eox013 (PMC5631097; doi:10.1093/emph/eox013)

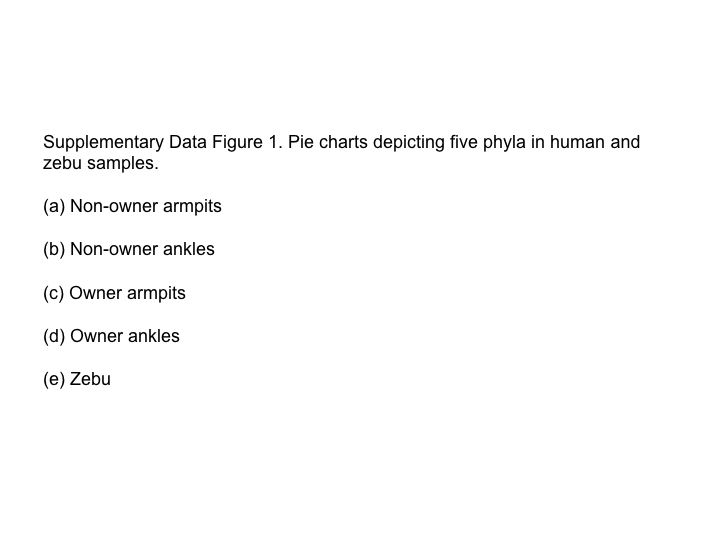

Supplement: Supplementary Figure_Legends [file eox013_Supp_Figure_legend.png]

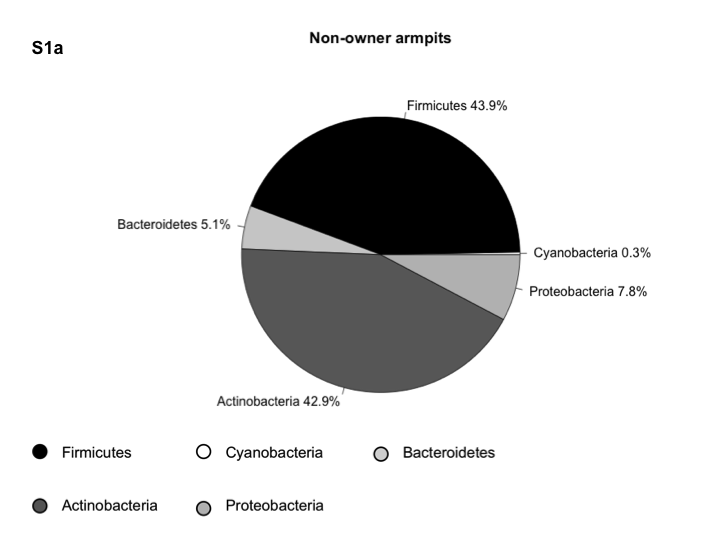

Supplement: eox013_Supp_Figure1a [file eox013_Supp_Figure1a.png]

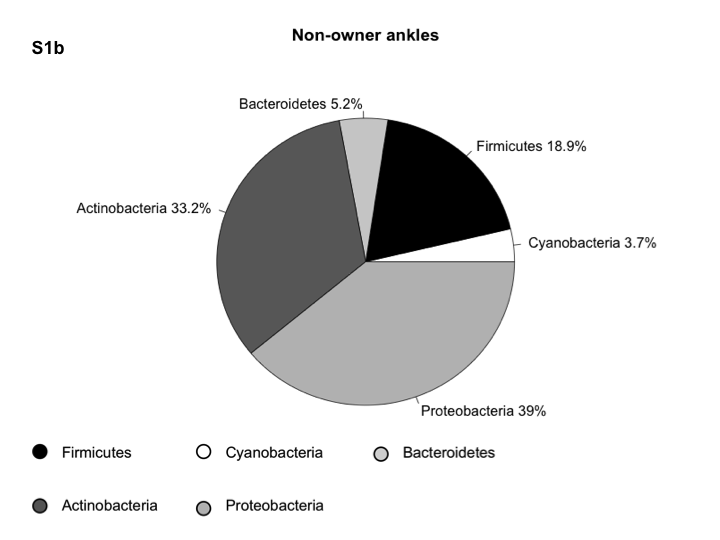

Supplement: eox013_Supp_Figure1b [file eox013_Supp_Figure1b.png]

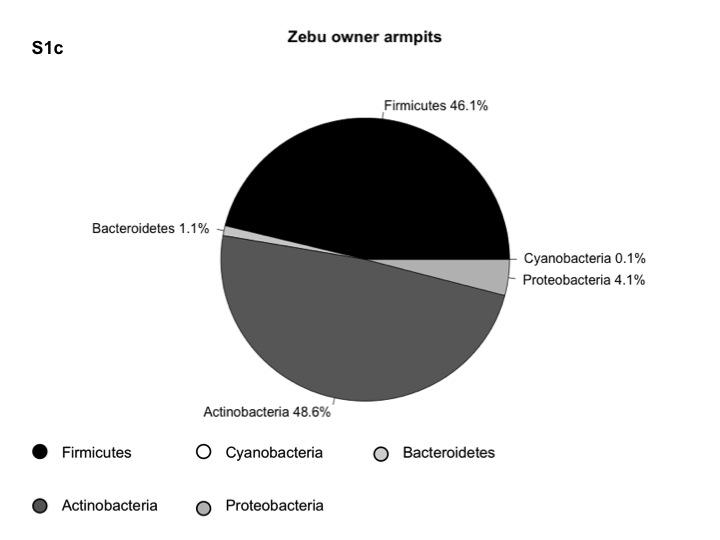

Supplement: eox013_Supp_Figure1c [file eox013_Supp_Figure1c.png]

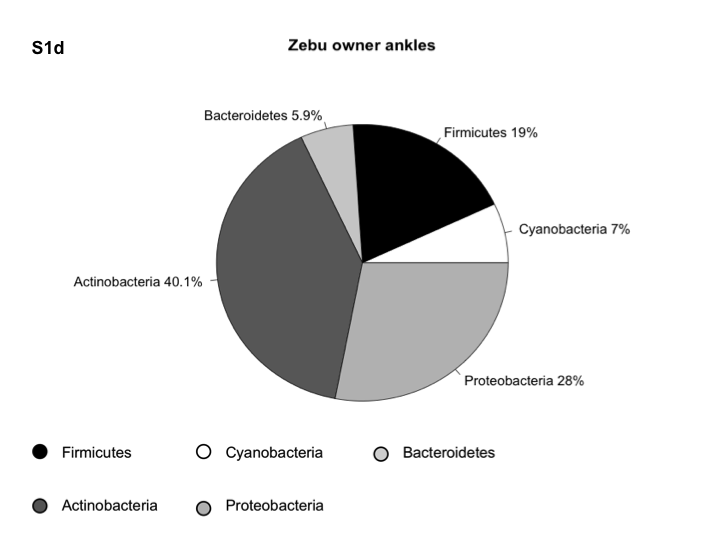

Supplement: eox013_Supp_Figure1d [file eox013_Supp_Figure1d.png]

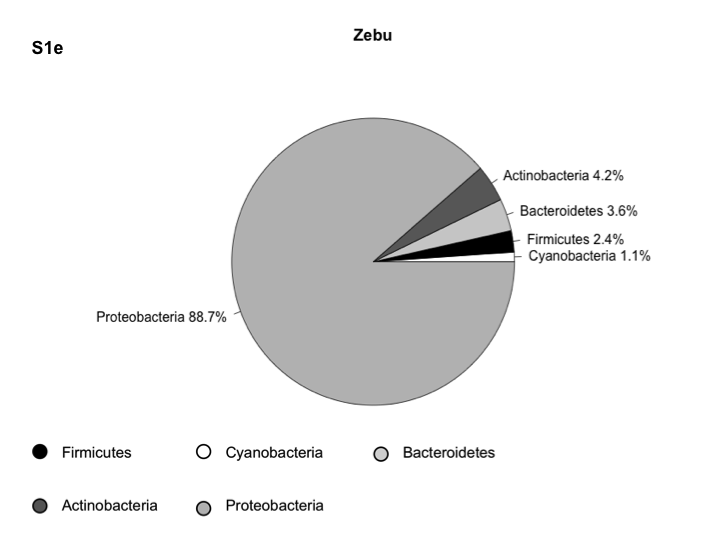

Supplement: eox013_Supp_Figure1e [file eox013_Supp_Figure1e.png]
